# Development of cerebral microhemorrhages in a mouse model of hypertension

**DOI:** 10.1186/s12974-025-03378-7

**Published:** 2025-03-05

**Authors:** Danny F. Xie, Chuo Fang, Christian Crouzet, Yu-Han Hung, Adrian Vallejo, Donghy Lee, Jihua Liu, Han Liu, Suhrith Muvvala, Annlia Paganini-Hill, Wei Ling Lau, David H. Cribbs, Bernard Choi, Mark Fisher

**Affiliations:** 1 Beckman Laser Institute and Medical Clinic, University of California, Irvine, CA USA; 2 Department of Biomedical Engineering, University of California, Irvine, CA USA; 3 Department of Neurology, University of California, Irvine, CA USA; 4 Institute for Memory Impairments and Neurological Disorders, University of California, Irvine, CA USA; 5 Department of Medicine, Division of Nephrology, University of California, Irvine, CA USA; 6 Department of Surgery, University of California, Irvine, CA USA; 7 Edwards Lifesciences Foundation Cardiovascular Innovation Research Center, University of California, Irvine, CA USA; 8 Department of Pathology & Laboratory Medicine, University of California, Irvine, CA USA

**Keywords:** Cerebral microhemorrhages, Cerebral microbleeds, Aging, Hypertension, Angiotensin II, Telmisartan, Microglial activation

## Abstract

Cerebral microhemorrhages (CMH) are the pathological substrate for MRI-demonstrable cerebral microbleeds, which are associated with cognitive impairment and stroke. Aging and hypertension are the main risk factors for CMH. In this study, we investigated the development of CMH in a mouse model of aging and hypertension. Hypertension was induced in aged (17-month-old) female and male C57BL/6J mice via angiotensin II (Ang II), a potent vasoconstrictor. We investigated the vascular origin of CMH using three-dimensional images of 1-mm thick brain sections. We examined Ang II-induced CMH formation with and without telmisartan, an Ang II type 1 receptor (AT1R) blocker. To evaluate the effect of microglia and perivascular macrophages on CMH formation, mice were treated with PLX3397, a selective colony-stimulating factor 1 receptor (CSF1R) inhibitor, to achieve microglial and macrophage depletion. Iba-1 and CD206 labeling were used to study the relative contributions of microglia and macrophages, respectively, on CMH formation. CMH quantification was performed with analysis of histological sections labeled with Prussian blue. Vessels surrounding CMH were primarily of capillary size range (< 10 μm in diameter). Ang II-infused mice exhibited elevated blood pressure (p < 0.0001) and CMH burden (p < 0.001). CMH burden was significantly correlated with mean arterial pressure in mice with and without Ang II (r = 0.52, p < 0.05). Ang II infusion significantly increased Iba-1 immunoreactivity (p < 0.0001), and CMH burden was significantly correlated with Iba-1 in mice with and without Ang II (r = 0.32, p < 0.05). Telmisartan prevented elevation of blood pressure due to Ang II infusion and blocked Ang II-induced CMH formation without affecting Iba-1 immunoreactivity. PLX3397 treatment reduced Iba-1 immunoreactivity in Ang II-infused mice (p < 0.001) and blocked Ang II-induced CMH (p < 0.0001). No significant association between CMH burden and CD206 reactivity was observed. Our findings demonstrate Ang II infusion increases CMH burden. CMH in this model appear to be capillary-derived and Ang II-induced CMH are largely mediated by blood pressure. In addition, microglial activation may represent an alternate pathway for CMH formation. These observations emphasize the continuing importance of blood pressure control and the role of microglia in hemorrhagic cerebral microvascular disease.

**Supplementary Information:**

The online version contains supplementary material available at 10.1186/s12974-025-03378-7.

## Introduction

Cerebral microhemorrhages (CMH) are the pathological substrate for cerebral microbleeds (CMB), chronic hemorrhages appearing as small foci of hemosiderin deposits on magnetic resonance imaging (MRI) [1]. CMB are associated with advanced age and found in almost 40% of those over 80 years old [2]. CMB have important implications for cognitive impairment and ischemic and hemorrhagic stroke [3–6].

CMB are typically found in elderly individuals with vascular comorbidities, especially hypertension [7, 8]. Hypertensive microangiopathy is known for the presence of microhemorrhages in the deep and infratentorial regions of the brain [2]. Clinical studies have shown an association between higher blood pressure levels and CMB in hypertensive patients [9, 10].

Angiotensin II (Ang II), a component of the renin-angiotensin system, binds to angiotensin II type 1 receptor (AT1R) and triggers several signaling pathways that increase blood pressure by causing vascular constriction and sympathetic nervous system stimulation [11–13]. Clinical studies have demonstrated the beneficial effect of reducing circulating angiotensinogen, a precursor of angiotensin, to lower blood pressure in patients with hypertension [14, 15]. Ang II-induced hypertension can lead to the generation of reactive oxygen species (ROS) in the cerebral microvasculature [16], which can negatively impact blood–brain barrier integrity [17, 18]. Additionally, activation of AT1R has been demonstrated to shift microglia towards an activated, proinflammatory phenotype [19]. Higher levels of inflammatory markers have also been observed in individuals with CMB [20]. Hypertension and neuroinflammation, two downstream effects of the AT1R pathway, may contribute to CMH formation, yet the relative role of each condition to CMH burden remains unknown.

The vascular origin of CMH, with and without hypertension, remains unclear [21]. To study the origin further, a reliable method of visualizing CMH and the surrounding cerebral microvasculature is necessary [22]. Standard histology has limitations in identifying the relationship between CMH and blood vessels due to the reliance on individual, thin (4–40 µm) brain sections collected at sparse intervals [23], which limits detailed visualization of CMH in context with the complex three-dimensional cerebral microvascular network.

Here we investigated the role of Ang II and the AT1R pathway in the formation of CMH by incorporating telmisartan, an AT1R blocker. We further investigated the role of microglia and macrophages in Ang II-induced CMH formation by incorporating PLX3397 treatment, a selective colony-stimulating factor 1 receptor (CSF1R) inhibitor that depletes microglia and macrophages. To study the vascular origin of CMH, we used a previously developed tissue clearing method (iDISCO) with lectin-DyLight as an endothelial marker to visualize the cerebral microvasculature alongside CMH in 1-mm thick brain sections [24, 25].

## Materials and methods

### Animal model and experimental design

Aged (17-month-old) C57BL/6J female and male mice (NIA Aged Rodent Colonies) were used for all experiments. Mice were acclimated for one week before experiments and had free access to food and water. All experimental procedures were conducted in accordance with the NIH Guide for the Care and Use of Laboratory Animals and were approved by the Institutional Animal Care and Use Committee at the University of California, Irvine. Systolic and diastolic blood pressure (mmHg) were measured before Alzet pump implantation (Baseline) and at the end of each study (Final) using a tail-cuff system (CODA, Kent Scientific, Torrington, CT) as described previously [26].

In Experiment 1, mice were randomly assigned to two experimental groups (n = 10 per sex per group): (1) Control and (2) Angiotensin II. To induce hypertension, Ang II (Bachem, Torrance, CA, USA) was infused into the mice at a rate of 1000 ng/kg/min using Alzet subcutaneous osmotic pumps (Alzet® Model 2004, Durect Corporation, Cupertino, CA, USA) for 4 weeks as described previously [26]. Control mice received Alzet pump implants that infused a vehicle (PBS).

In Experiment 2, mice were randomly assigned to four experimental groups (n = 12–15 per sex per group): (1) Control, (2) Angiotensin II, (3) Control + Telmisartan, and (4) Angiotensin II + Telmisartan. Telmisartan (Cayman Chemical Company, Ann Arbor, Michigan, USA) was dissolved in a 1 mL solution of 1 N sodium hydroxide, neutralized with 1 N hydrochloric acid, and administered at 0.5 mg/kg/day in the drinking water for 4 weeks.

In Experiment 3, mice were randomly assigned to four experimental groups (n = 3–5 per sex per group): (1) Control, (2) Angiotensin II, (3) Control + PLX3397, and (4) Angiotensin II + PLX3397. To deplete microglia and macrophages, we used a highly selective inhibitor PLX3397 (pexidartinib, MedChemExpress, New Jersey, USA), which targets the CSF1R [27–30]. PLX3397 was administered in the form of AIN-76A standard chow at a concentration of 290 mg/kg (Dyets, Inc., PA, USA). The mice were given the PLX3397 diet for a total of 7 weeks, starting 3 weeks before and continuing for 4 weeks during the infusion of Ang II or PBS.

### Detection and quantification of CMH

Four weeks following Alzet pump implantation, mice were anesthetized using inhaled isoflurane and underwent cardiac perfusion with ice-cold PBS for 5 min. The brains were then collected, bisected into hemibrains, fixed overnight in 4% paraformaldehyde (Thermo Fisher Scientific, Cleveland, OH, USA), and prepared for histological analysis as described previously [31, 32]. Hemosiderin deposition, a marker of CMH, was detected using Prussian blue staining. Brains were sectioned into coronal sections (20- or 40-µm) with a freezing microtome (Thermo Fisher Scientific, Cleveland, OH, USA), and every seventh section was stained with a solution containing 5% potassium hexacyanoferrate trihydrate (Sigma-Aldrich, St. Louis, MO, USA) and 10% hydrochloric acid (Sigma-Aldrich, St. Louis, MO, USA). After 30 min, the sections were rinsed in water, counterstained with nuclear fast red, dehydrated, and cover-slipped (Research Services Core, Department of Pathology and Laboratory Medicine at UCI Medical Center). CMH were identified and photographed under a light microscope at 20 × magnification by a reader blinded to experimental groups. Whole slides were scanned and analyzed to determine the total area of the brain section. Automated quantification of CMH area (µm^2^) was performed by a blinded reader using a ratiometric approach based on the segmentation ratio of the red/green/blue pixel intensities [33]. The average number of CMH was quantified and adjusted relative to the total brain surface area analyzed per animal [34].

### Immunostaining for Iba-1 and CD206

To detect neuroinflammation, sections (20- or 40-µm) were prepared and treated as follows: For Iba-1, we used 1% bovine serum albumin as a blocking agent for 2 h at room temperature. We then incubated the sections with rabbit primary antibodies against Iba-1 (microglia/macrophage marker) (Wako Chemicals USA, Richmond, VA, USA) at 1:400 dilution overnight at 4 °C, washed with PBST, and incubated with a biotinylated anti-rabbit IgG secondary antibody (1:1000 dilution, Jackson ImmunoResearch, West Grove, PA, USA) for two hours at room temperature and with avidin–biotin-peroxidase (ABC) complex (Vector Laboratories, Burlingame, CA, USA) for 45 min at room temperature. Staining was performed using 3,3′-diaminobenzidine (DAB) (Vector Laboratories, Burlingame, CA, USA) following the manufacturer's instructions. A reader blinded to the experimental groups captured and analyzed three images at 20 × magnification from each of three brain regions (cortex, hippocampus, and thalamus). The immunoreactive area was quantified as a percentage of the total analyzed area using NIH ImageJ software 1.52 [34].

For CD206 (to detect perivascular macrophages), we used 10% donkey serum as a blocking agent for 1 h at room temperature, incubated with rat primary antibodies against CD206 (macrophage marker) (Bio-Rad Laboratories, Hercules, CA, USA) at 1:500 dilution overnight at 4 °C, washed with PBST, and then incubated with anti-rat IgG secondary antibody (Thermo Fisher Scientific, Waltham, MA, USA) at 1:200 dilution for 1 h at room temperature. CD206-labeled brain sections were imaged using a Leica True Confocal Scanner SP8 (Leica Biosystems, Illinois, USA) with a 10x/0.3 NA HC PL Fluotar objective. The imaging system used a 485–488 nm laser to image the CD206 bound to the perivascular macrophages, with emission captured between 510 and 570 nm. A reader blinded to the experimental groups captured images of the entire brain section. The immunoreactive area was quantified as the ratio of the immunofluorescent area to total brain cortex area. Analysis was focused on the brain cortex because perivascular macrophages are primarily present along cortical vessels.

### Optical clearing for vascular localization of cerebral microhemorrhages

Cerebral microvasculature was labeled with lectin-DyLight-649. Before euthanasia, mice were injected retro-orbitally with a 200 µL solution of lectin-DyLight-649 diluted in saline (0.25 mg/mL) (Vector Laboratories, Burlingame, CA, USA). The solution was allowed to circulate for 20 min. Cardiac perfusion was then performed with phosphate-buffered saline (PBS) for 5 min at 2 mL/min, followed by perfusion with 10% formalin for 5 min at 2 mL/min. Hemibrains were drop-fixed overnight in 10% formalin. Hemibrains were then cut with a vibratome into 1-mm thick coronal sections. Prussian blue staining was done using 5% potassium hexacyanoferrate trihydrate (Sigma-Aldrich, St. Louis, MO, USA) and 10% hydrochloric acid (Sigma-Aldrich, St. Louis, MO, USA). After 60 min, sections were rinsed in water. Sections were cleared following a modified iDISCO (immunolabeling-enabled three-dimensional imaging of solvent-cleared organs) protocol [24, 25, 35]. Briefly, samples were dehydrated in a series of 20-min methanol washes (20%, 40%, 60%, 80%, 100%, and 100%) and immersion in a solution of 66% dichloromethane and 33% methanol for 1 h. Finally, sections were immersed in dichloromethane twice for 15 min and stored in dibenzyl ether for refractive index matching.

All brain sections (6 per hemibrain) were imaged using a Leica True Confocal Scanner SP8 (Leica Biosystems, Illinois, USA) with a 10x/0.3 NA HC PL Fluotar objective. The imaging system used a 633 nm HeNe laser to image the lectin-DyLight-649 bound to the brain vasculature, with emission captured between 638 and 783 nm. Simultaneously, a transmission image was taken to visualize Prussian blue-positive CMH. Sections were scanned at 10 × magnification using a light microscope by a reader blinded to experimental groups for Prussian blue-positive tissue regions. At these tissue regions, stacks of images were collected that cover the entire thickness of the brain section.

Vascular localization was performed using custom-written scripts in MATLAB and the neuTube open-source neuron tracing software [36]. First, we employed an iterative selection thresholding method [37] in MATLAB for automated image segmentation. Then, we utilized neuTube to trace the segmented vasculature, allowing us to determine blood vessel diameters at specific locations. Additionally, images of CMH were segmented using a Sauvola thresholding method [38]. This segmentation facilitated simple identification of the centroid of each Prussian-blue positive region that constitutes a CMH. We recorded the diameter of the five nearest vessels to each identified centroid.

### Statistical analysis

Results are presented as means ± SEM. Differences among experimental groups were tested using one-way ANOVA or two-way ANOVA with Tukey multiple comparisons. Pearson correlation coefficients were calculated to assess associations between variables. Differences and correlations were considered statistically significant if a two-tailed p-value < 0.05. The data were analyzed using GraphPad Prism 8 (GraphPad Software, La Jolla, CA).

## Results

### Vasculature surrounding CMH is primarily in the capillary size range

**Fig. 1:**
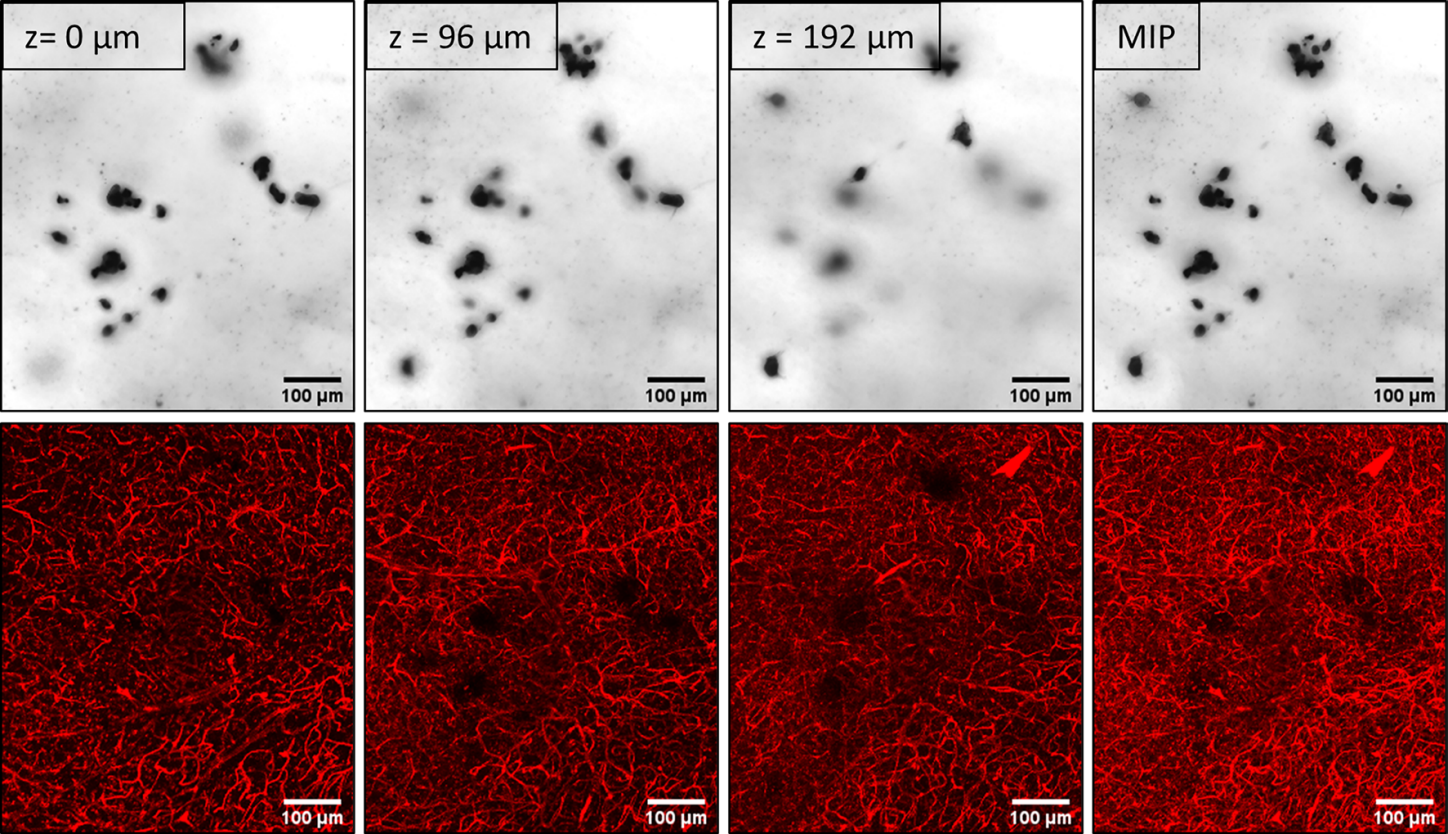
Three-dimensional microscopy of cleared, thick brain sections enables simultaneous visualization of CMH and cerebral vasculature. Top row: Confocal transmission images of Prussian-blue positive deposits. Bottom row: Confocal fluorescence images of lectin-Dylight-649 labeled brain vasculature. Regions with no visible vessels correspond to locations where a Prussian-blue positive deposit is present. Images displayed represent various depths within a CMH. This example shows a CMH extending across a tissue region ~ 200 µm thick. The rightmost image is a maximum intensity projection of the entire tissue region. Scale bars are 100 μm

**Fig. 2:**
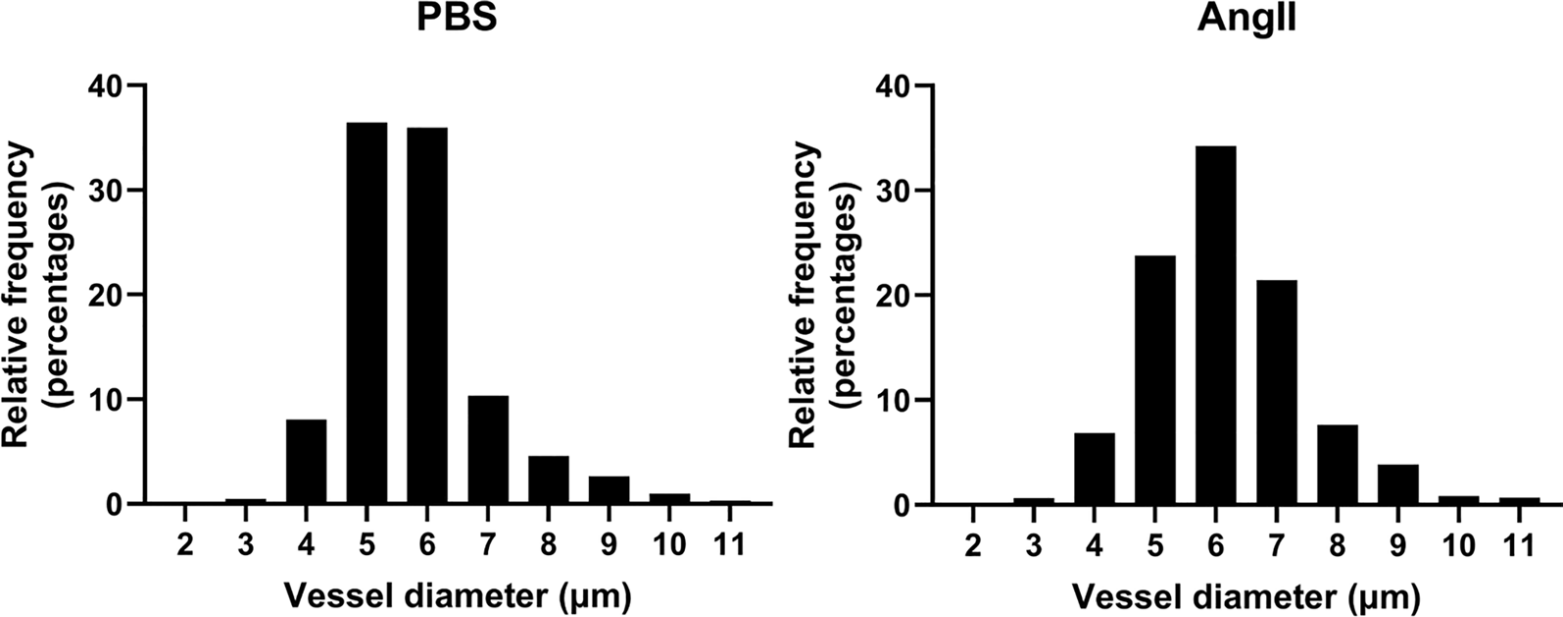
The majority of vessels nearest to a CMH have inner diameters less than 10 µm. For each Prussian-blue positive deposit, the diameters of the five nearest vessels to the centroid of each deposit were quantified. This finding suggests that CMH formation primarily occurs near capillaries

In mice treated with Ang II or PBS (Experiment 1), we examined tissue regions containing a CMH lesion and the surrounding vasculature **(**Fig. 1**)**. We focused on candidate blood vessels located nearest to each Prussian blue-positive deposit that form a single CMH lesion. In both groups, inner lumen diameters were between 2–11 µm, and the majority (> 97%) of the identified vessels had a diameter below 10 µm **(**Fig. 2**)**.

### Ang II produced elevated blood pressure and increased formation of CMH

**Fig. 3:**
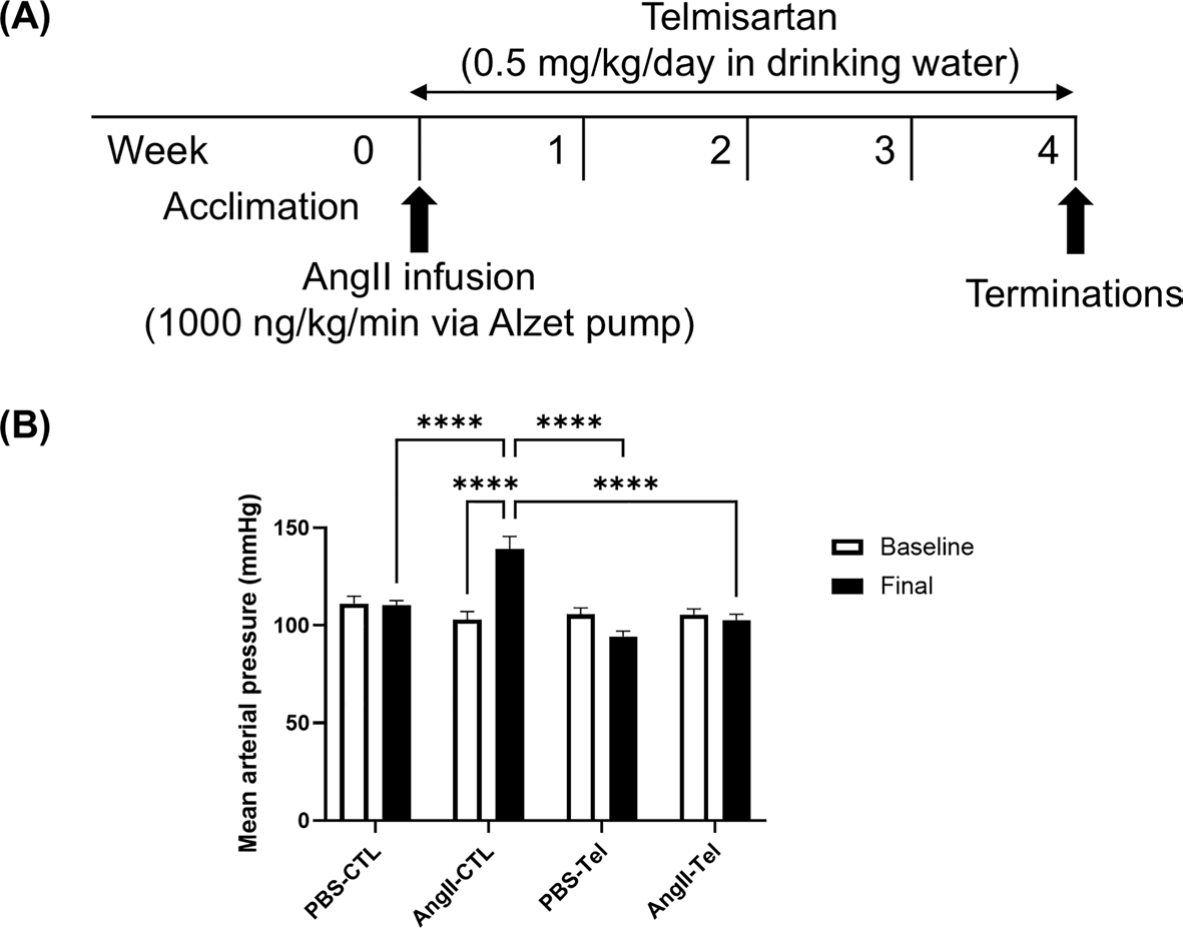
Angiotensin II infusion significantly increased mean arterial pressure, and this was eliminated by telmisartan administration. (**A**) The experimental timeline involved a 4-week infusion of Ang II (1000 ng/kg/min) or a control vehicle (PBS) via an Alzet pump. Telmisartan, a blocker of the Ang II type 1 receptor (AT1R), was administered at 0.5 mg/kg/day in drinking water for the same duration. (**B**) Blood pressure measurements (mmHg) were taken before and after the pump implantation using the tail-cuff technique. These data indicate a clear effect of telmisartan in blocking Ang II-induced hypertension in this mouse model. Data shown are mean ± SEM. n = 12–15 per sex per group. ****p < 0.0001

**Fig. 4:**
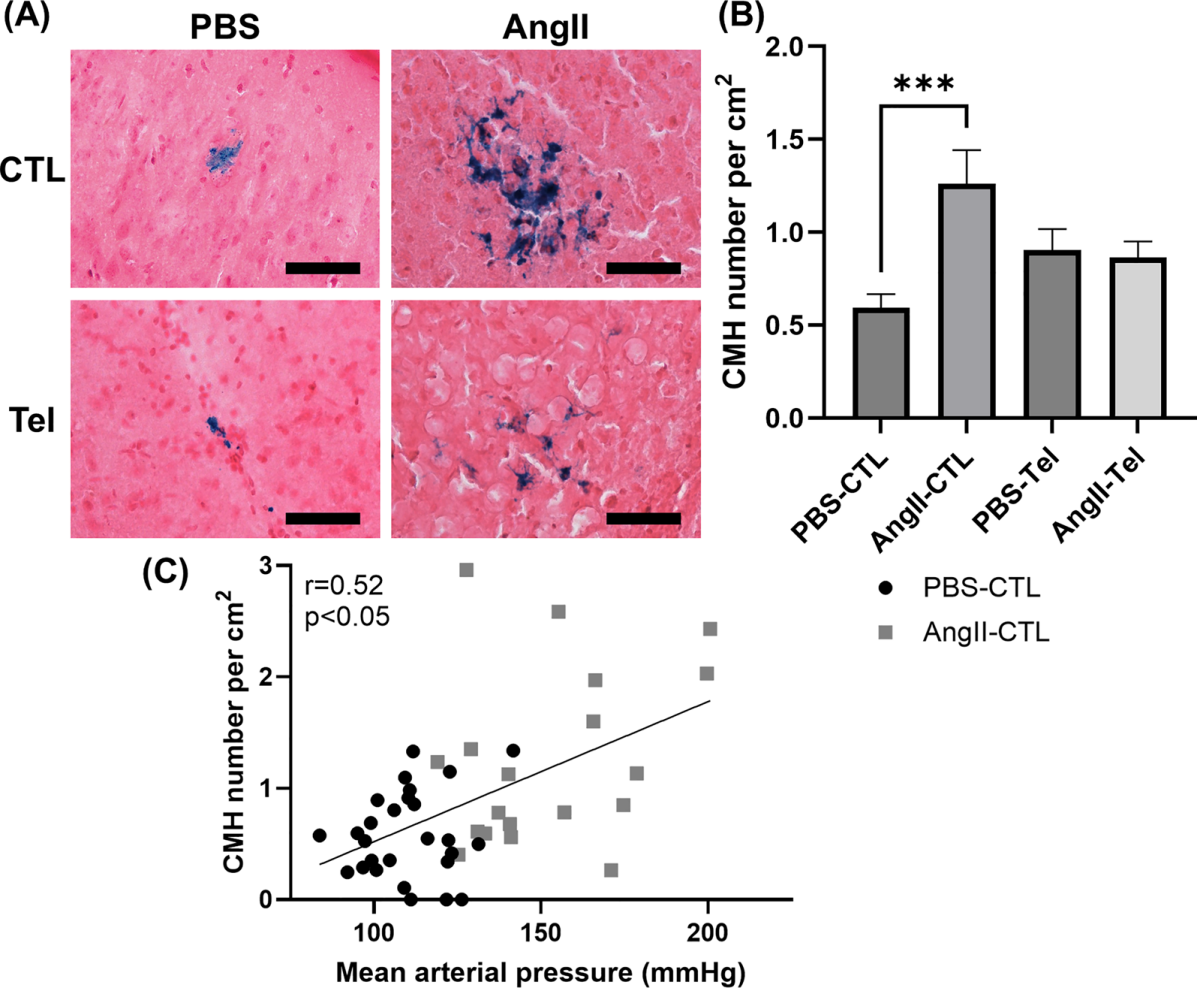
Ang II infusion promotes CMH formation. (**A**) Images showing Prussian blue-positive deposits, indicative of CMH. Color balance was adjusted for visualization. Analysis was performed on raw images. Scale bar = 100 µm. (**B**) The number of Prussian blue-positive deposits, indicating CMH, was significantly higher in mice with Ang II infusion compared with control mice with PBS infusion, while telmisartan blocked AngII-induced increase in CMH (AngII-Tel vs PBS-Tel). (**C**) In animals with and without Ang II-induced hypertension (PBS-CTL and AngII-CTL), the number of CMH was positively correlated with MAP (r = 0.52). Data shown are mean ± SEM. n = 12–15 per sex per group. ***p < 0.001

In Experiment 2, Ang II infusion for 4 weeks **(**Fig. 3A**)** significantly increased mean arterial pressure (MAP) (Baseline: 103 ± 4 mmHg to Final: 139 ± 6 mmHg, p < 0.0001) **(**Fig. 3B**)**. CMH were identified as regions positive for Prussian blue (Fig. 4A). The number of CMH was 2.1 times higher in mice with Ang II-induced hypertension compared with controls (AngII-CTL: 1.26 ± 0.18 per cm^2^ vs. PBS-CTL: 0.59 ± 0.07 per cm^2^, p < 0.001) **(**Fig. 4B**)**. In mice with and without Ang II infusion, the number of CMH was positively correlated with MAP (r = 0.52, p < 0.05) **(**Fig. 4C**)**. The number of CMH events is comparable to what was observed using a different mouse model of CMH [39]. The number of CMH did not differ between male mice and female mice within each group (Supplemental Fig. S1). A majority (70%) of CMH were located in the subcortex brain region (Supplemental Fig. S3).

### Telmisartan prevented Ang II-induced blood pressure elevation and reduced Ang II-induced CMH formation

Telmisartan, an AT1R blocker, effectively prevented MAP elevation in mice with Ang II infusion (Baseline: 106 ± 3 to Final: 102 ± 3 mmHg, p > 0.05) **(**Fig. 3B**)**. In mice treated with telmisartan, there was no increase in CMH number in response to Ang II infusion. (PBS-Tel: 0.91 ± 0.11 per cm^2^ to AngII-Tel: 0.86 ± 0.09 per cm^2^, p > 0.05) **(**Fig. 4B**)**.

### Ang II infusion led to elevated Iba-1 immunoreactivity

**Fig. 5:**
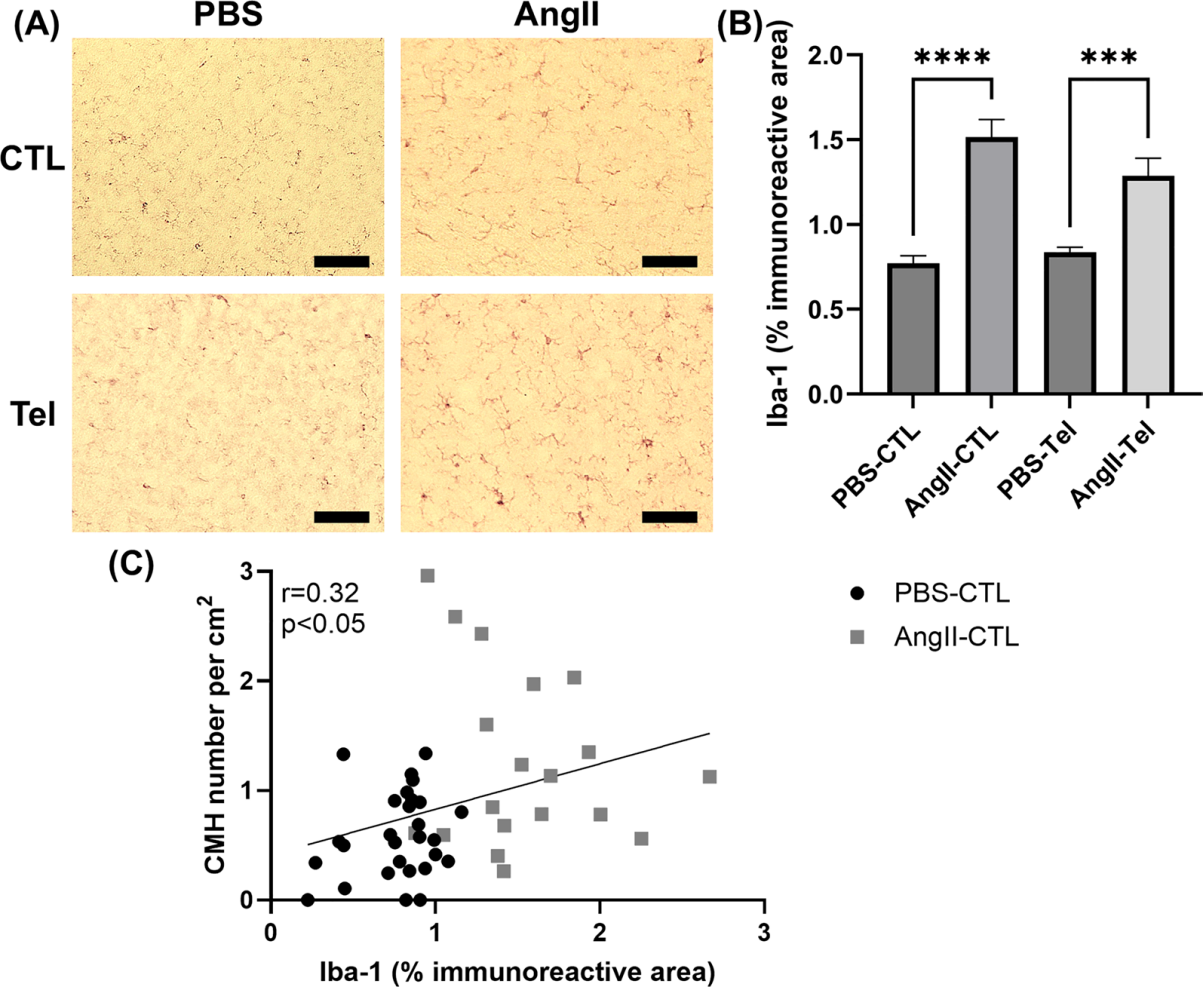
Ang II infusion increases Iba-1 immunoreactivity. (**A**) Images of Iba-1 immunohistochemistry. Color balance was adjusted for visualization. Analysis was performed on raw images. Scale bar = 100 µm. (**B**) Immunoreactivity of Iba-1, a marker for microglia and macrophages, was significantly higher in Ang II-infused mice. Telmisartan treatment did not affect Iba-1 immunoreactivity. (**C**) In animals with and without Ang II-induced hypertension (PBS-CTL and AngII-CTL), the number of CMH was positively correlated with Iba-1 immunoreactivity (r = 0.32). Data shown are mean ± SEM. n = 12–15 per sex per group. ***p < 0.001 and ****p < 0.0001

Immunohistochemistry with Iba-1 revealed that mice with Ang II-induced hypertension had a two-fold increase in Iba-1 immunoreactivity compared with the control group (AngII-CTL: 1.52 ± 0.10% vs. PBS-CTL: 0.77 ± 0.05%, p < 0.0001) (Fig. 5A, B). Iba-1 immunoreactivity remained elevated in telmisartan- and AngII-treated mice (AngII-Tel: 1.29 ± 0.10% vs. PBS-Tel: 0.84 ± 0.03%, p < 0.0001). (Fig. 5A, B). In mice with and without Ang II infusion, the number of CMH was positively correlated with Iba-1 immunoreactivity (r = 0.32, p < 0.05) **(**Fig. 5C**)**.

### Ang II-infusion led to elevated blood pressure with and without PLX3397 diet

**Fig. 6:**
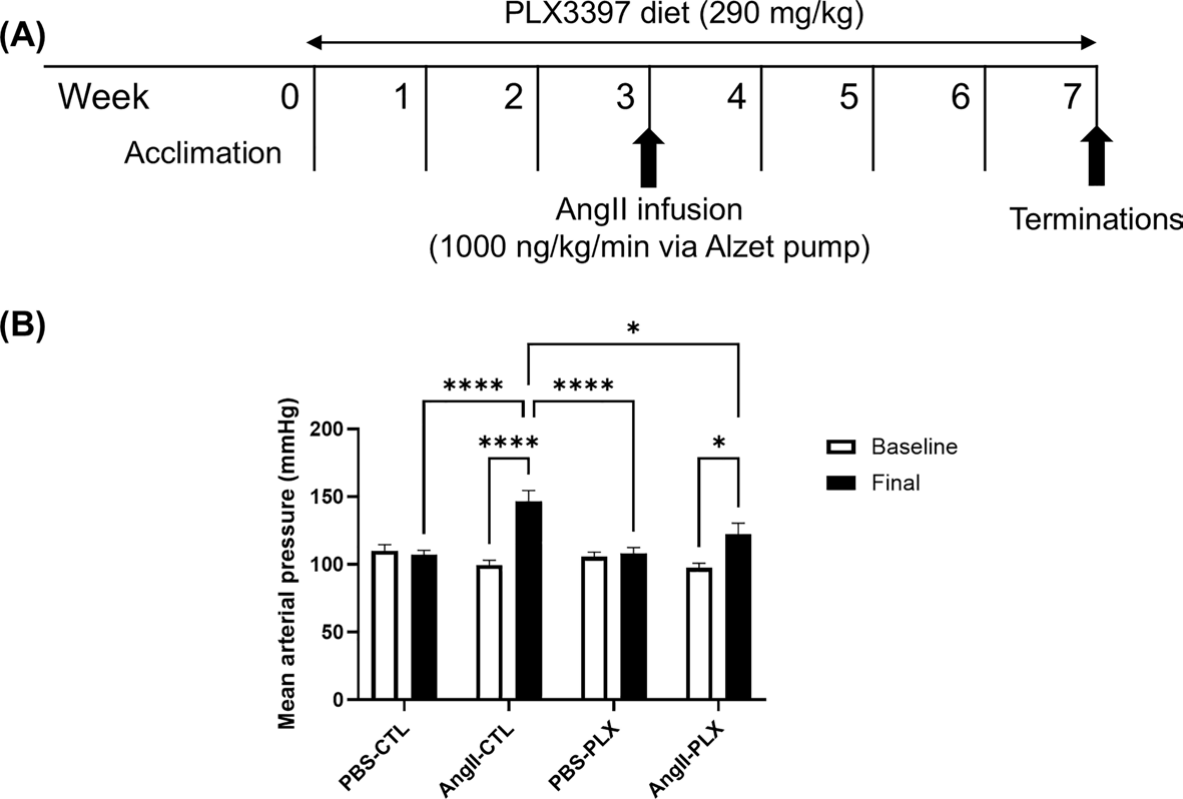
Ang II infusion led to elevated blood pressure with and without PLX3397 diet. (**A**) In this study, the effect of microglial depletion on Ang II-induced CMH formation was examined. Mice were given a PLX3397 diet for 7 weeks, including 3 weeks before and 4 weeks during hypertension induction with Ang II infusion (1000 ng/kg/min). (**B**) Both regular chow and PLX3397 diet groups showed an increase in mean arterial pressure due to Ang II infusion. Final MAP was lower in mice with Ang II and PLX3397 than mice with Ang II alone. Data shown are mean ± SEM. n = 3–5 per sex per group. *p < 0.05 and ****p < 0.0001

In Experiment 3, we investigated the relationship between microglial/macrophage activation and Ang II-induced CMH formation. Ang II infusion for 4 weeks **(**Fig. 6A**)** elevated MAP in both regular chow-fed (Baseline: 99 ± 4 mmHg to Final: 147 ± 8 mmHg, p < 0.0001) and PLX3397-fed (Baseline: 98 ± 3 mmHg to Final: 122 ± 8 mmHg p < 0.05) groups (Fig. 6B). In mice with PBS infusion, the PLX3397 diet did not affect blood pressure levels (Fig. 6B). The final blood pressure was lower in mice with Ang II infusion and PLX3397 diet than mice with Ang II infusion alone (AngII-PLX: 122 ± 8 mmHg vs. AngII-CTL: 147 ± 8 mmHg, p < 0.05) (Fig. 6B).

### Iba-1 immunoreactivity was significantly reduced by PLX3397 diet

**Fig. 7:**
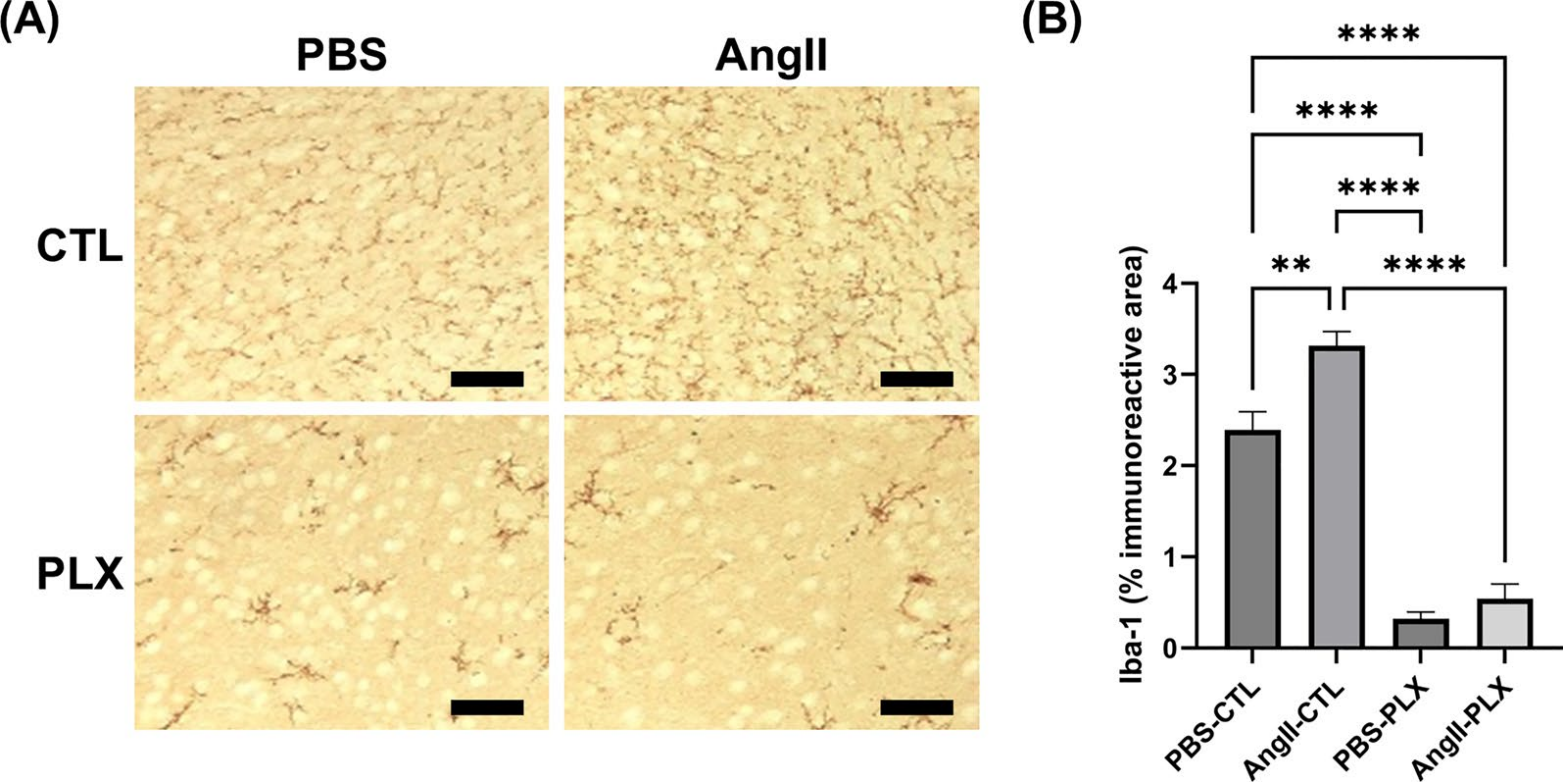
PLX3397 diet significantly reduced Iba-1 immunoreactivity. (**A**) Images of Iba-1 immunohistochemistry. Color balance was adjusted for visualization. Analysis was performed on raw images. Scale bar = 100 µm. (**B**) Immunoreactivity of Iba-1 was significantly lower in mice on the PLX3397 diet than those on regular chow. The increase in immunoreactivity induced by Ang II was eliminated in mice on the PLX3397 diet. Data shown are mean ± SEM. n = 3–5 per sex per group. **p < 0.01 and ****p < 0.0001

Significantly lower Iba-1 immunoreactivity was observed in mice on the PLX3397 diet compared with regular chow-fed mice. The increase in Iba-1 immunoreactivity induced by Ang II was absent in mice fed the PLX3397 diet (AngII-PLX: 0.54 ± 0.16% vs. AngII-CTL: 3.32 ± 0.15%, p < 0.001) (Fig. 7B). Mice with Ang II infusion had a larger proportion of activated microglia and a smaller proportion of resting microglia, suggesting a shift to the activated state (Supplemental Fig. S4).

### PLX3397 diet inhibited the formation of CMH induced by Ang II

**Fig. 8:**
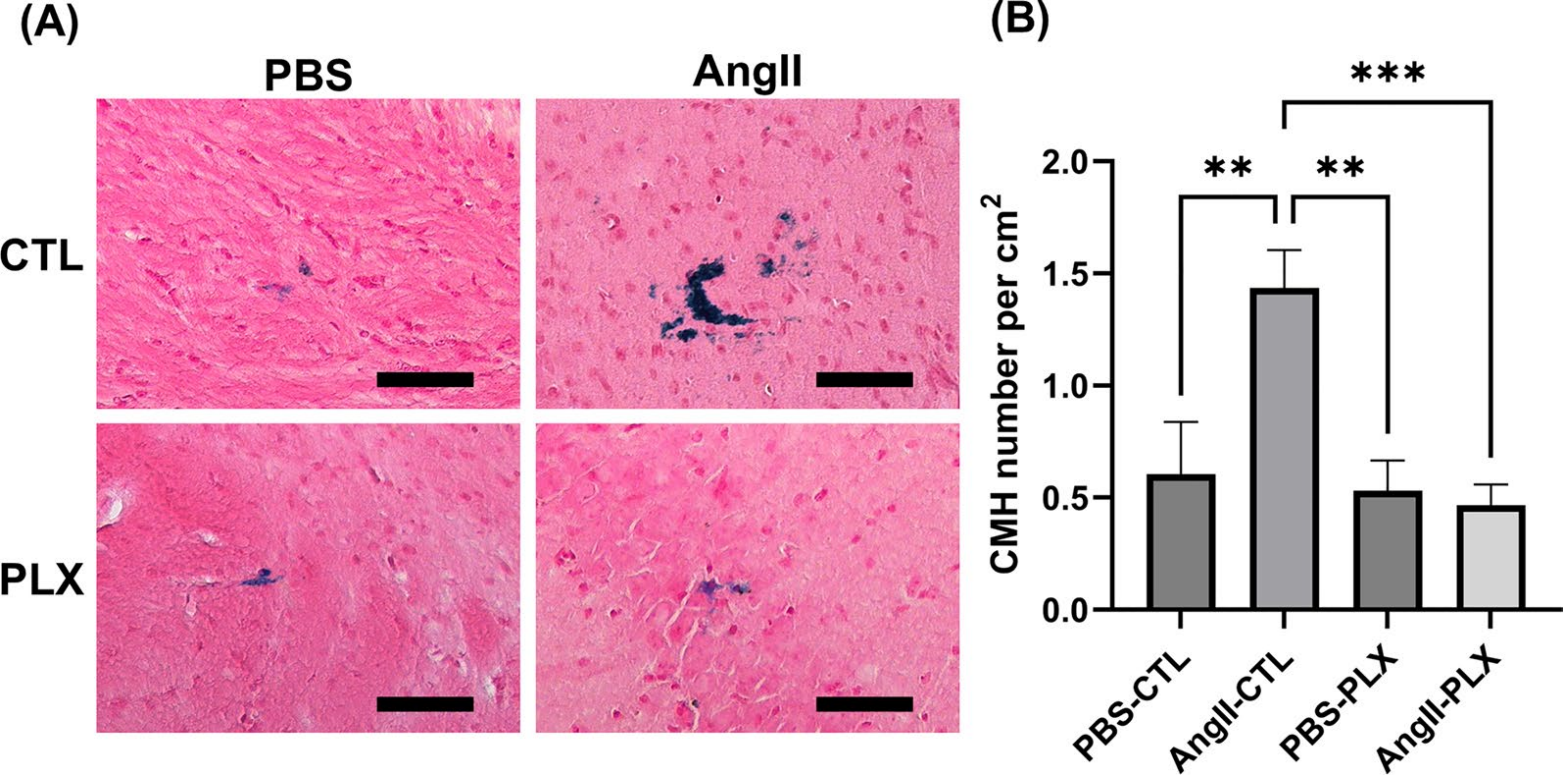
PLX3397 diet significantly reduced CMH number in mice with Ang II-induced hypertension. (**A**) Images showing Prussian blue-positive deposits, indicative of CMH. Color balance was adjusted for visualization. Analysis was performed on raw images. Scale bar = 100 µm. (**B**) As shown previously, CMH number per cm^2^ is elevated in response to Ang II infusion. However, PLX3397 diet inhibited the formation of CMH induced by Ang II. Data shown are mean ± SEM. n = 3–5 per sex per group. **p < 0.01 and ***p < 0.001

The PLX3397 diet significantly reduced the Ang II-induced CMH number (AngII-CTL: 1.44 ± 0.47 per cm^2^ to AngII-PLX: 0.47 ± 0.10 per cm^2^, p < 0.0001), bringing it to a level comparable to untreated animals in the PBS-CTL group (Fig. 8B). The number of CMH was not significantly different between female mice and male mice within each group (data not shown).

### CD206 immunoreactivity is unaffected by Ang II and is significantly reduced by PLX3397 diet

**Fig. 9:**
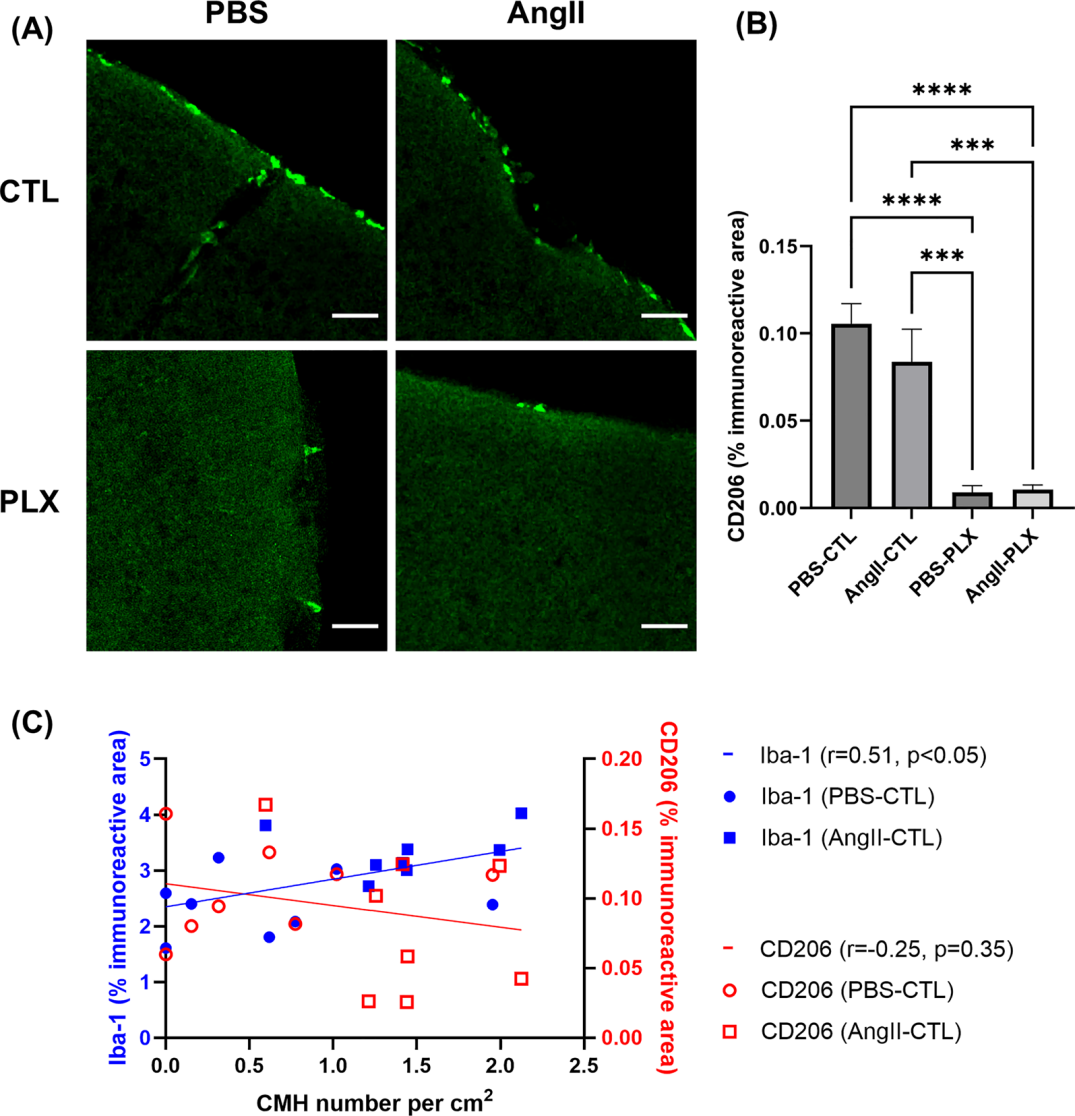
CD206 immunoreactivity is unaffected by Ang II and is significantly reduced by PLX3397 diet. (**A**) Images showing CD206 immunofluorescence, indicative of perivascular macrophages. Scale bar = 50 µm. (**B**) Ang II infusion does not affect CD206 immunoreactivity, while PLX3397 diet significantly reduces CD206 immunoreactivity. (**C**) In animals with and without Ang II-induced hypertension (PBS-CTL and AngII-CTL), the number of CMH was positively correlated with Iba-1 immunoreactivity (r = 0.51). A nonsignificant (p = 0.35) association was observed between CD206 immunoreactivity and CMH number per cm^2^. Data shown are mean ± SEM. n = 3–5 per sex per group. ***p < 0.001 and ****p < 0.0001

To distinguish the impact of microglia and macrophages on CMH formation, we used CD206, a marker of perivascular macrophages (Fig. 9A) [40]. Our results demonstrate that Ang II infusion did not affect CD206 immunoreactivity (Fig. 9B). However, PLX3397 diet reduced CD206 immunoreactivity (Fig. 9B). We then compared the relationship of Iba-1 immunoreactivity and CD206 immunoreactivity with CMH formation (Fig. 9C) in non-PLX diet mice (PBS-CTL and AngII-CTL). A significant correlation was observed between CMH number and Iba-1 immunoreactivity (r = 0.51, p < 0.05); no significant association was observed between CMH number and CD206 immunoreactivity (r = -0.25, p = 0.35).

## Discussion

The role of hypertension as a risk factor for CMH and its underlying mechanisms have been unclear. Here, we induced hypertension in aged C57BL/6J mice by infusing Ang II, a potent vasoconstrictor component of the renin–angiotensin–aldosterone system (RAAS). We investigated the vascular origin of CMH and determined that capillaries are the predominant vessel type surrounding CMH. We quantified blood pressure, CMH count, and indices of neuroinflammation (Iba-1 and CD206 immunoreactivity) to study the relationship among these factors. Ang II-infused mice exhibited elevated blood pressure, elevated Iba-1 immunoreactivity, and higher CMH count. Telmisartan eliminated this blood pressure increase and prevented CMH formation. Microglial depletion significantly reduced both CMH count and Iba-1 immunoreactivity in Ang II-induced hypertension, while mitigating the hypertensive effect of Ang II. Finally, Ang II infusion had no effect on CD206 immunoreactivity, a macrophage-specific marker, and CD206 reactivity was not associated with CMH count. Collectively, our findings demonstrate hypertension-induced CMH formation and suggest microglial activation as a possible alternate pathway to CMH formation.

Ang II plays a major role in the RAAS in regulating blood pressure and blood volume [12, 41]. This is achieved by inducing vasoconstriction and increasing outflow from the sympathetic nervous system [42, 43]. In our experiments, infusion of Ang II over a 28-day period resulted in increased MAP, indicating successful hypertension induction with Ang II (Fig. 3B). Mice with Ang II infusion had a higher CMH count than mice without Ang II infusion (Fig. 4B), consistent with prior work [21], and the increased CMH was directly associated with extent of hypertension (Fig. 4C). Collectively, these results indicate that CMH formation occurs via a hypertension-dependent mechanism [44].

The extent of hypertension did not differ between female and male mice. This finding is different from other published studies that show female mice are less susceptible to Ang II-induced hypertension, possibly due to protective effects from estrogen [45, 46]. The present study used aged mice (18-months-old) in which lower estrogen levels occur, as it is reported that 80% of female mice have irregular or absent estrus cycling by 17-months of age [47]. Our data imply that by 17- to 18-months of age, female mice have little or no estrogen protection against effects of Ang II.

CMH number did not differ between female and male mice. In our previous study using adenine to induce chronic kidney disease (CKD) model to induce CMH formation, CMH number was larger in male mice than in female mice [39], perhaps due to higher serum creatinine observed in male mice and increased susceptibility to adenine-induced CKD. Individual pathways for CMH formation may thus be sex-dependent or sex-independent.

Previous studies have investigated the potential vascular source of CMH using Prussian blue-stained iron deposits with standard histological sections. Some findings suggest CMH appear primarily around arterioles [48, 49] while other findings suggest CMH appear primarily around capillaries [32, 50]. Here, we used tissue clearing to collect three-dimensional imaging of Prussian-blue stained CMH along with the surrounding fluorescently labeled vasculature (Fig. 1). We found that the majority (97%) of microvessels surrounding a CMH had a diameter less than 10 μm **(**Fig. 2**)**, suggesting that capillaries are the primary vascular source of CMH in this model.

To modulate the effects of Ang II on blood pressure, we administrated telmisartan, an AT1R blocker that can prevent Ang II effects such as vasoconstriction [51]. Telmisartan is frequently used as a drug to treat hypertension [52]. In the present study, we observed that telmisartan prevented Ang II-induced elevation of blood pressure (Fig. 3B). Telmisartan treatment was effective in blocking Ang II-induced CMH formation (Fig. 4B). Collectively, these results suggest that eliminating hypertension can reduce CMH burden.

Telmisartan did not impact neuroinflammation (Fig. 5B) despite the known presence of AT1R on microglia [53, 54]. This may be explained by the organ-specific penetration of telmisartan [55, 56]. A study investigating the distribution of telmisartan in the body found that some organs such as the intestine, kidneys, liver, and heart absorb more than five times the amount of telmisartan compared with the brain [56]. Data on brain penetration of AT1R blockers suggest low penetration of telmisartan into the brain [55]. This suggests a high dosage of telmisartan may be needed to achieve a significant effect on the brain, but a lower dosage of telmisartan may be sufficient to impact systemic factors such as blood pressure.

Ang II binding to AT1 receptors leads to activation of NADPH oxidase, which results in the production of reactive oxygen species (ROS) and inflammatory processes. ROS are involved with the activation of microglia [57]. Microglial activation has been shown to occur in hypertensive mice, and microglia may contribute to sustaining hypertension [58]. Our findings are consistent with these observations, with Ang II-infused mice having elevated levels of Iba-1 immunoreactivity (Fig. 5B). This effect may specifically result from the interaction of Ang II with AT1R expressed by microglia [19]. Investigations using non-Ang II hypertension models may further clarify the relationship between blood pressure and microglial activation.

Ang II-induced neurovascular dysfunction independent of blood pressure elevation has been reported previously [59, 60]. To modulate neuroinflammation, we incorporated the PLX3397 diet (pexidartinib), an inhibitor of the CSF1R. The CSF1R is expressed by macrophages and microglia, and activation of this receptor is required for the proliferation and survival of macrophages and microglia [27]. Depletion of microglia can attenuate the blood pressure elevation induced by Ang II [58, 61], which is consistent with the current study in which we observed a lesser severity of hypertension in mice with microglial depletion (Fig. 6B). Microglial and macrophage depletion was successfully achieved (Fig. 7B), and a reduction in Ang II-induced CMH number was observed with microglial and macrophage depletion (Fig. 8B). Collectively, these findings suggest microglial involvement in CMH formation, but further studies are required to investigate this phenomenon.

**Fig. 10:**
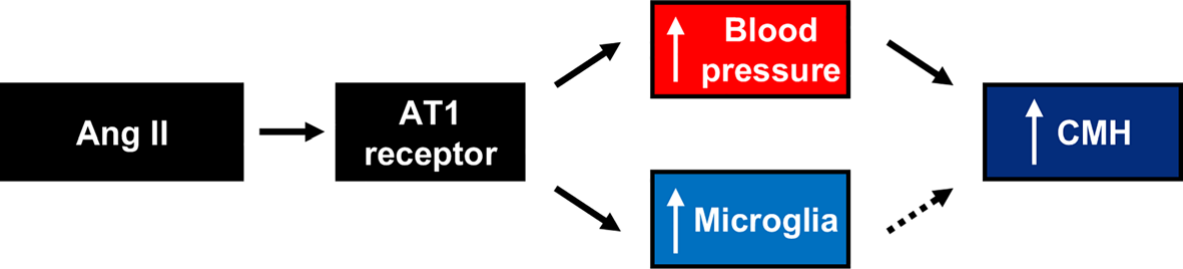
Schematic of the relationship between Ang II and CMH. Ang II infusion leads to hypertension and microglial activation. Increased blood pressure results in increase CMH formation, while microglial activation may also lead to CMH

To distinguish between the contribution of microglia and macrophages to CMH formation, we labeled brain sections with CD206, a marker of perivascular macrophages. Consistent with prior work [62], Ang II infusion did not affect CD206 immunoreactivity and PLX3397 diet significantly reduced CD206 immunoreactivity (Fig. 9B). Furthermore, we observed a significant correlation between CMH number and Iba-1 immunoreactivity (r = 0.51, p < 0.05) which was not present between CMH number and CD206 immunoreactivity. These findings imply that microglia are the main type of myeloid cell contributing to Ang II-induced CMH formation. Collectively, our results suggest that hypertension is a major contributor of CMH formation, and CMH burden can be reduced by normalizing blood pressure or microglial depletion (Fig 10)

Our study has limitations. We relied on diameter measurements for the classification of vessel types [63]. The iDISCO clearing method can lead to tissue shrinkage, reducing surface area by about 30% [64]. Within our results, a 30% reduction in surface area can lead to an error in a diameter measurement by approximately 2 µm. In addition, we did not directly measure blood–brain barrier permeability and its potential role in CMH formation in CMH formation in this model. We cannot address whether our findings are specific for Ang II-induced hypertension, an issue that deserves further study.

## Conclusions

Ang II infusion in aged mice leads to hypertension and microglial activation. Our data demonstrate a hypertension-dependent pathway of CMH formation, which primarily appear around capillary-sized vessels. Our data also suggest a microglial-dependent pathway for CMH formation that is relatively independent of hypertension, but further studies are required. These findings highlight that in addition to hypertension control, microglia may be a novel therapeutic target in CMH prevention.

## Supplementary Information

Additional file 1.

## Data Availability

The datasets used and/or analyzed during the current study are available from the corresponding author upon reasonable request.

## Abbreviations

Ang II: Angiotensin II
AT1R: Angiotensin II type 1 receptor
CMB: Cerebral microbleeds
CMH: Cerebral microhemorrhages
CSF1R: Colony-stimulating factor 1 receptor
iDISCO: Immunolabeling-enabled three-dimensional imaging of solvent-cleared organs
PBS: Phosphate-buffered saline
PBST: Phosphate-buffered saline with Tween
RAAS: Renin–angiotensin–aldosterone system
ROS: Reactive oxygen species

## References

[1] Haller S, Vernooij MW, Kuijer JPA, Larsson EM, Jäger HR, Barkhof F. Cerebral microbleeds: Imaging and clinical significance. Radiology. 2018;287(1):11–28. 10.1148/radiol.2018170803. 29558307 10.1148/radiol.2018170803

[2] Vernooij MW, Van Der Lugt A, Ikram MA, Wielopolski PA, Niessen WJ, Hofman A, et al. Prevalence and risk factors of cerebral microbleeds: the Rotterdam scan study. Neurology. 2008;70(14):1208–14. 10.1212/01.wnl.0000307750.41970.d9. 18378884 10.1212/01.wnl.0000307750.41970.d9

[3] Akoudad S, Wolters FJ, Viswanathan A, De Bruijn RF, Van Der Lugt A, Hofman A, et al. Association of cerebral microbleeds with cognitive decline and dementia. JAMA Neurol. 2016;73(8):934–43. 10.1001/jamaneurol.2016.1017. 27271785 10.1001/jamaneurol.2016.1017 PMC5966721

[4] Wilson D, Charidimou A, Ambler G, Fox ZV, Gregoire S, Rayson P, et al. Recurrent stroke risk and cerebral microbleed burden in ischemic stroke and TIA. Neurology. 2016;87(14):1501–10. 10.1212/WNL.0000000000003183. 27590288 10.1212/WNL.0000000000003183 PMC5075978

[5] Bokura H, Saika R, Yamaguchi T, Nagai A, Oguro H, Kobayashi S, et al. Microbleeds are associated with subsequent hemorrhagic and ischemic stroke in healthy elderly individuals. Stroke. 2011;42(7):1867–71. 10.1161/STROKEAHA.110.601922. 21597015 10.1161/STROKEAHA.110.601922

[6] Tsushima Y, Aoki J, Endo K. Brain microhemorrhages detected on T2*-weighted gradient-echo MR images. AJNR Am J Neuroradiol. 2003;24(1):88–96. 12533332 PMC8148967

[7] Elmståhl S, Ellström K, Siennicki-Lantz A, Abul-Kasim K. Association between cerebral microbleeds and hypertension in the Swedish general population “Good Aging in Skåne” study. J Clin Hypertens. 2019;21(8):1099–107. 10.1111/jch.13606. 10.1111/jch.13606 PMC6771849 31274244

[8] Reddy ST, Savitz SI. Hypertension-related cerebral microbleeds. Case Rep Neurol. 2020;12(3):266–9. 10.1159/000508760. 33082763 10.1159/000508760 PMC7548923

[9] Van Dooren M, Staals J, De Leeuw PW, Kroon AA, Henskens LH, Van Oostenbrugge RJ. Progression of brain microbleeds in essential hypertensive patients: A 2-year follow-up study. Am J Hypertens. 2014;27(8):1045–51. 10.1093/ajh/hpu032. 24610885 10.1093/ajh/hpu032

[10] Lyu L, Shen J, Zeng C, Ji J, Hu W, Wei T, et al. Cerebral microbleeds are associated with blood pressure levels in individuals with hypertension. Clin Exp Hypertens. 2020;42(4):328–34. 10.1080/10641963.2019.1665673. 31542967 10.1080/10641963.2019.1665673

[11] Kawai T, Forrester SJ, O’Brien S, Baggett A, Rizzo V, Eguchi S. AT1 receptor signaling pathways in the cardiovascular system. Pharmacol Res. 2017;125:4–13. 10.1016/j.phrs.2017.05.008. 28527699 10.1016/j.phrs.2017.05.008 PMC5607088

[12] Fyhrquist F, Metsarinne K, Tikkanen I. Role of angiotensin II in blood pressure regulation and in the pathophysiology of cardiovascular disorders. J Hum Hypertens. 1995;9(5):S19-24. 8583476

[13] Kinzenbaw DA, Langmack L, Faraci FM. Angiotensin II-induced endothelial dysfunction: Impact of sex, genetic background, and rho kinase. Physiol Rep. 2022;10(11):e15336. 10.1481/phy2.15336. 35681278 10.14814/phy2.15336 PMC9184751

[14] Bakris GL, Saxena M, Gupta A, Chalhoub F, Lee J, Stiglitz D, et al. RNA interference with zilebesiran for mild to moderate hypertension the KARDIA-1 randomized clinical trial. JAMA. 2024;331(9):740–9. 10.1001/jama.2024.0728. 38363577 10.1001/jama.2024.0728 PMC10873804

[15] Schiffrin EL. RNA injection every 6 months to improve adherence and lower blood pressure in patients with hypertension. JAMA. 2024;331(9):733–5. 10.1001/jama.2023.26071. 38363578 10.1001/jama.2023.26071

[16] Girouard H, Park L, Anrather J, Zhou P, Iadecola C. Angiotensin II attenuates endothelium-dependent responses in the cerebral microcirculation through nox-2-derived radicals. Arterioscler Thromb Vasc Biol. 2006;26(4):826–32. 10.1161/01.ATV.0000205849.22807.6e. 16439707 10.1161/01.ATV.0000205849.22807.6e

[17] Bell RD, Zlokovic BV. Neurovascular mechanisms and blood-brain barrier disorder in Alzheimer’s disease. Acta Neuropathol. 2009;118(1):103–13. 10.1007/s00401-009-0522-3. 19319544 10.1007/s00401-009-0522-3 PMC2853006

[18] Gella A, Durany N. Oxidative stress in Alzheimer disease. Cell Adhes Migr. 2009;3(1):88–93. 10.4161/cam.3.1.7402. 10.4161/cam.3.1.7402 PMC2675154 19372765

[19] Phipps JA, Vessey KA, Brandli A, Nag N, Tran MX, Jobling AI, et al. The Role of Angiotensin II/AT1 Receptor Signaling in Regulating Retinal Microglial Activation. Invest Ophthalmol Vis Sci. 2018;59(1):487–98. 10.1167/iovs.17-22416. 29368003 10.1167/iovs.17-22416

[20] Miwa K, Tanaka M, Okazaki S, Furukado S, Sakaguchi M, Kitagawa K. Relations of blood inflammatory marker levels with cerebral microbleeds. Stroke. 2011;42(11):3202–6. 10.1161/STROKEAHA.111.621193. 21868735 10.1161/STROKEAHA.111.621193

[21] Toth P, Tarantini S, Springo Z, Tucsek Z, Gautam T, Giles CB, et al. Aging exacerbates hypertension-induced cerebral microhemorrhages in mice: role of resveratrol treatment in vasoprotection. Aging Cell. 2015;14(3):400–8. 10.1111/acel.12315. 25677910 10.1111/acel.12315 PMC4406669

[22] Lin E, Alessio A. What are the basic concepts of temporal, contrast, and spatial resolution in cardiac CT? J Cardiovasc Comput Tomogr. 2009;3(6):403–8. 10.1016/j.jcct.2009.07.003. 19717355 10.1016/j.jcct.2009.07.003 PMC4752333

[23] Liang X, Luo H. Optical tissue clearing: illuminating brain function and dysfunction. Theranostics. 2021;11(7):3035–51. 10.7150/THNO.53979. 33537072 10.7150/thno.53979 PMC7847687

[24] Khouri K, Xie DF, Crouzet C, Bahani AW, Cribbs DH, Fisher MJ, et al. Simple methodology to visualize whole-brain microvasculature in three dimensions. Neurophotonics. 2021;8(02):1–10. 10.1117/1.NPh.8.2.025004. 10.1117/1.NPh.8.2.025004 PMC8056070 33884280

[25] Xie DF, Crouzet C, LoPresti K, Wang Y, Robinson C, Jones W, et al. Semi-automated protocol to quantify and characterize fluorescent three-dimensional vascular images. PLoS ONE. 2024. 10.1371/journal.pone.0289109. 38753706 10.1371/journal.pone.0289109 PMC11098357

[26] Passos GF, Kilday K, Gillen DL, Cribbs DH, Vasilevko V. Experimental hypertension increases spontaneous intracerebral hemorrhages in a mouse model of cerebral amyloidosis. J Cereb Blood Flow Metab. 2016;36(2):399–404. 10.1177/0271678X15606720. 26661173 10.1177/0271678X15606720 PMC4759670

[27] Elmore MRP, Najafi AR, Koike MA, Dagher NN, Spangenberg EE, Rice RA, et al. Colony-stimulating factor 1 receptor signaling is necessary for microglia viability, unmasking a microglia progenitor cell in the adult brain. Neuron. 2014;82(2):380–97. 10.1016/j.neuron.2014.02.040. 24742461 10.1016/j.neuron.2014.02.040 PMC4161285

[28] Spangenberg EE, Lee RJ, Najafi AR, Rice RA, Elmore MRP, Blurton-Jones M, et al. Eliminating microglia in Alzheimer’s mice prevents neuronal loss without modulating amyloid-β pathology. Brain. 2016;139(4):1265–81. 10.1093/brain/aww016. 26921617 10.1093/brain/aww016 PMC5006229

[29] Sosna J, Philipp S, Albay RI, Reyes-Ruiz JM, Baglietto-Vargas D, LaFerla FM, et al. Early long-term administration of the CSF1R inhibitor PLX3397 ablates microglia and reduces accumulation of intraneuronal amyloid, neuritic plaque deposition and pre-fibrillar oligomers in 5XFAD mouse model of Alzheimer’s disease. Mol Neurodegener. 2018;13(1):11. 10.1186/s13024-018-0244-x. 29490706 10.1186/s13024-018-0244-x PMC5831225

[30] Kerkhofs D, Van Hagen BT, Milanova IV, Schell KJ, Van Essen H, Wijnands E, et al. Pharmacological depletion of microglia and perivascular macrophages prevents vascular cognitive impairment in Ang II-induced hypertension. Theranostics. 2020;10(21):9512–27. 10.7150/thno.44394. 32863942 10.7150/thno.44394 PMC7449902

[31] Sumbria RK, Grigoryan MM, Vasilevko V, Paganini-Hill A, Kilday K, Kim R, et al. Aging exacerbates development of cerebral microbleeds in a mouse model. J Neuroinflammation. 2018;15(1):1–7. 10.1186/s12974-018-1092-x. 29510725 10.1186/s12974-018-1092-x PMC5840821

[32] Sumbria RK, Grigoryan MM, Vasilevko V, Krasieva TB, Scadeng M, Dvornikova AK, et al. A murine model of inflammation-induced cerebral microbleeds. J Neuroinflammation. 2016;13(1):1–12. 10.1186/s12974-016-0693-5. 27577728 10.1186/s12974-016-0693-5 PMC5006574

[33] Crouzet C, Jeong G, Chae RH, LoPresti KT, Dunn CE, Xie DF, et al. Spectroscopic and deep learning-based approaches to identify and quantify cerebral microhemorrhages. Sci Rep. 2021;11(1):10725. 10.1038/s41598-021-88236-1. 34021170 10.1038/s41598-021-88236-1 PMC8140127

[34] Lau WL, Nunes ACF, Vasilevko V, Floriolli D, Lertpanit L, Savoj J, et al. Chronic kidney disease increases cerebral microbleeds in mouse and man. Transl Stroke Res. 2020;11(1):122–34. 10.1007/s12975-019-00698-8. 31055735 10.1007/s12975-019-00698-8 PMC6957561

[35] Renier N, Wu Z, Simon DJ, Yang J, Ariel P, Tessier-Lavigne M. IDISCO: A simple, rapid method to immunolabel large tissue samples for volume imaging. Cell. 2014;159(4):896–910. 10.1016/j.cell.2014.10.010. 25417164 10.1016/j.cell.2014.10.010

[36] Feng L, Zhao T, Kim J. Neutube 1.0: a new design for efficient neuron reconstruction software based on the swc format. ENeuro. 2015. 10.1523/ENEURO.0049-14.2014. 26464967 10.1523/ENEURO.0049-14.2014 PMC4586918

[37] Ridler TW, Calvard S. Picture thresholding using an iterative slection method. IEEE Trans Syst Man Cybern. 1978;8(8):630–2.

[38] Sauvola J, Pietikäinen M. Adaptive document image binarization. Pattern Recognit. 2000;33(2):225–36. 10.1016/S0031-3203(99)00055-2.

[39] Fang C, Lau WL, Sun J, Chang R, Vallejo A, Lee D, et al. Chronic kidney disease promotes cerebral microhemorrhage formation. J Neuroinflammation. 2023;20(1):51. 10.1186/s12974-023-02703-2. 36841828 10.1186/s12974-023-02703-2 PMC9960195

[40] Goldmann T, Wieghofer P, Jordão MJC, Prutek F, Hagemeyer N, Frenzel K, et al. Origin, fate and dynamics of macrophages at central nervous system interfaces. Nat Immunol. 2016;17(7):797–805. 10.1038/ni.3423. 27135602 10.1038/ni.3423 PMC4968048

[41] Fountain JH, Lappin SL. Physiology renin angiotensin system. Florida: StatPearls; 2018. 29261862

[42] Reid IA. Interactions between ANG II, sympathetic nervous system, and baroreceptor reflexes in regulation of blood pressure. Am J Physiol - Endocrinol Metab. 1992. 10.1152/ajpendo.1992.262.6.e763. 10.1152/ajpendo.1992.262.6.E763 1616014

[43] Harrison-Bernard LM. The renal renin-angiotensin system. Am J Physiol - Adv Physiol Educ. 2009;33(4):270–4. 10.1152/advan.00049.2009. 10.1152/advan.00049.2009 19948673

[44] Pacholko A, Iadecola C. Hypertension, neurodegeneration, and cognitive decline. Hypertension. 2024;81(5):991–1007. 10.1161/HYPERTENSIONAHA.123.21356. 38426329 10.1161/HYPERTENSIONAHA.123.21356 PMC11023809

[45] Faraci FM, Lamping KG, Modrick ML, Ryan MJ, Sigmund CD, Didion SP. Cerebral vascular effects of angiotensin II: New insights from genetic models. J Cereb Blood Flow Metab. 2006;26(4):449–55. 10.1038/sj.jcbfm.9600204. 16094317 10.1038/sj.jcbfm.9600204

[46] Xue B, Pamidimukkala J, Hay M. Sex differences in the development of angiotensin II-induced hypertension in conscious mice. Am J Physiol Circ Physiol. 2005;288(5):H2177–84. 10.1152/ajpheart.00969.2004. 10.1152/ajpheart.00969.2004 15626687

[47] Frick KM, Burlingame LA, Arters JA, Berger-Sweeney J. Reference memory, anxiety and estrous cyclicity in C57BL/6NIA mice are affected by age and sex. Neuroscience. 2000;95(1):293–307. 10.1016/s0306-4522(99)00418-2. 10619486 10.1016/s0306-4522(99)00418-2

[48] Janaway BM, Simpson JE, Hoggard N, Highley JR, Forster G, Drew D, et al. Brain haemosiderin in older people: Pathological evidence for an ischaemic origin of magnetic resonance imaging (MRI) microbleeds. Neuropathol Appl Neurobiol. 2014;40(3):258–69. 10.1111/nan.12062. 23678850 10.1111/nan.12062 PMC4282337

[49] Lo P, Crouzet C, Vasilevko V, Choi B. Visualization of microbleeds with optical histology in mouse model of cerebral amyloid angiopathy. Microvasc Res. 2016;105:109–13. 10.1016/j.mvr.2016.02.002. 26876114 10.1016/j.mvr.2016.02.002 PMC4814270

[50] Fisher M, French S, Ji P, Kim RC. Cerebral microbleeds in the elderly: a pathological analysis. Stroke. 2010;41(12):2782–5. 10.1161/STROKEAHA.110.593657. 21030702 10.1161/STROKEAHA.110.593657 PMC3079284

[51] Bakheit AHH, Abd-Elgalil AA, Mustafa B, Haque A, Wani TA. Telmisartan. Profiles Drug Subst Excipients Relat Methodol. 2015;40:371–429. 10.1016/bs.podrm.2015.01.003. 10.1016/bs.podrm.2015.01.003 26051689

[52] Sharpe M, Jarvis B, Goa KL. Telmisartan: a review of its use in hypertension. Drugs. 2001;61(10):1501–29. 10.2165/00003495-200161100-00009. 11558835 10.2165/00003495-200161100-00009

[53] Biancardi VC, Stranahan AM, Krause EG, de Kloet AD, Stern JE. Cross talk between AT 1 receptors and Toll-like receptor 4 in microglia contributes to angiotensin II-derived ROS production in the hypothalamic paraventricular nucleus. Am J Physiol Circ Physiol. 2016;310(3):H404–15. 10.1152/ajpheart.00247.2015. 10.1152/ajpheart.00247.2015 PMC4796625 26637556

[54] Mowry FE, Peaden SC, Stern JE, Biancardi VC. TLR4 and AT1R mediate blood-brain barrier disruption, neuroinflammation, and autonomic dysfunction in spontaneously hypertensive rats. Pharmacol Res. 2021. 10.1016/j.phrs.2021.105877. 34610452 10.1016/j.phrs.2021.105877 PMC8648989

[55] Michel MC, Foster C, Brunner HR, Liu L. A systematic comparison of the properties of clinically used angiotensin II type 1 receptor antagonists. Pharmacol Rev. 2013;65(2):809–48. 10.1124/pr.112.007278. 23487168 10.1124/pr.112.007278

[56] Shimizu K, Takashima T, Yamane T, Sasaki M, Kageyama H, Hashizume Y, et al. Whole-body distribution and radiation dosimetry of [11C]telmisartan as a biomarker for hepatic organic anion transporting polypeptide (OATP) 1B3. Nucl Med Biol. 2012;39(6):847–53. 10.1016/j.nucmedbio.2012.01.008. 22421430 10.1016/j.nucmedbio.2012.01.008

[57] Labandeira-Garcia JL, Rodríguez-Perez AI, Garrido-Gil P, Rodriguez-Pallares J, Lanciego JL, Guerra MJ. Brain renin-angiotensin system and microglial polarization: Implications for aging and neurodegeneration. Front Aging Neurosci. 2017. 10.3389/fnagi.2017.00129. 28515690 10.3389/fnagi.2017.00129 PMC5413566

[58] Shen XZ, Li Y, Li L, Shah KH, Bernstein KE, Lyden P, et al. Microglia participate in neurogenic regulation of hypertension. Hypertension. 2015;66(2):309–16. 10.1161/HYPERTENSIONAHA.115.05333. 26056339 10.1161/HYPERTENSIONAHA.115.05333 PMC4498964

[59] Kazama K, Anrather J, Zhou P, Girouard H, Frys K, Milner TA, et al. Angiotensin II impairs neurovascular coupling in neocortex through NADPH oxidase-derived radicals. Circ Res. 2004;95(10):1019–26. 10.1161/01.RES.0000148637.85595.c5. 15499027 10.1161/01.RES.0000148637.85595.c5

[60] Capone C, Faraco G, Park L, Cao X, Davisson RL, Iadecola C. The cerebrovascular dysfunction induced by slow pressor doses of angiotensin II precedes the development of hypertension. Am J Physiol Circ Physiol. 2011;300(1):H397-407. 10.1152/ajpheart.00679.2010. 10.1152/ajpheart.00679.2010 PMC3023263 20971763

[61] Wang M, Pan W, Xu Y, Zhang J, Wan J, Jiang H. Microglia-mediated neuroinflammation a potential target for the treatment of cardiovascular diseases. J Inflamm Res. 2022. 10.2147/JIR.S350109. 35642214 10.2147/JIR.S350109 PMC9148574

[62] Faraco G, Sugiyama Y, Lane D, Garcia-Bonilla L, Chang H, Santisteban MM, et al. Perivascular macrophages mediate the neurovascular and cognitive dysfunction associated with hypertension. J Clin Invest. 2016;126(12):4674–89. 10.1172/JCI86950. 27841763 10.1172/JCI86950 PMC5127678

[63] Sun J, Ou W, Han D, Paganini-Hill A, Fisher MJ, Sumbria RK. Comparative studies between the murine immortalized brain endothelial cell line (bEnd3) and induced pluripotent stem cell-derived human brain endothelial cells for paracellular transport. PLoS One. 2022. 10.1371/journal.pone.0268860. 35613139 10.1371/journal.pone.0268860 PMC9132315

[64] Bossolani GDP, Pintelon I, Detrez JD, Buckinx R, Thys S, Zanoni JN, et al. Comparative analysis reveals Ce3D as optimal clearing method for in toto imaging of the mouse intestine. Neurogastroenterol Motil. 2019;31(5):1–11. 10.1111/nmo.13560. 10.1111/nmo.13560 30761698

[65] Lier J, Streit WJ, Bechmann I. Beyond activation: characterizing microglial functional phenotypes. Cells. 2021. 10.3390/cells10092236. 34571885 10.3390/cells10092236 PMC8464670

[66] Crews FT, Vetreno RP. Mechanisms of neuroimmune gene induction in alcoholism. Psychopharmacology (Berl). 2016;233(9):1543–57. 10.1007/s00213-015-3906-1. 25787746 10.1007/s00213-015-3906-1 PMC4828484

