# Supplementary material for: Development of cerebral microhemorrhages in a mouse model of hypertension
Source: J Neuroinflammation. 2025 Mar 5;22:67. doi: 10.1186/s12974-025-03378-7 (PMC11881401; doi:10.1186/s12974-025-03378-7)
Supplement: Supplementary file 1 — Additional file 1. [file 12974_2025_3378_MOESM1_ESM.docx]

**Supplemental Data**

**Sex differences in CMH burden**

**Figure S1.** **CMH density (number per cm^2^) for female and male mice across all groups in Experiment 2.** No significant differences were observed between groups (α = 0.05). Data are presented as mean ± SEM, with n=12-15 per sex per group.

**Figure S2. CMH density (number per cm^2^) for female and male mice across all groups in Experiment 3.** No significant differences were observed between groups (α = 0.05). Data are presented as mean ± SEM, with n=12-15 per sex per group.

**CMH percentage by brain regions**

Each CMH was categorized into one of three brain regions: cortex, subcortex, and brainstem/cerebellum. The percentage of CMH in each brain region was calculated for each mouse. A repeated measures two-way ANOVA was used to evaluate: 1) differences in the CMH percentage across the three brain regions within a group and 2) differences in CMH percentage for a specific region between groups. The majority of CMH were located in the subcortex, with fewer in the cortex and brainstem/cerebellum regions. No significant differences were observed between Ang II-infused and control groups **(Fig. S3)**.

**Figure S3. CMH distribution across the cortex, subcortex, and brainstem/cerebellum regions in mice with and without Ang II-induced hypertension.** CMH are most abundant in the subcortex regardless of Ang II infusion. Data are presented as mean ± SEM, with n=12-15 per sex per group. ***p<0.001, ****p<0.0001.

**Microglial reactive state**

Coronal sections (20-um thick) were incubated in 0.3% hydrogen peroxide in 1 × PBS (pH 7.4) for 30 min at room temperature to block endogenous peroxidase activity. The sections were then blocked with PBS containing 0.3% of Triton X-100 (PBST) containing 5% donkey serum for 2 h at room temperature. They were then incubated overnight at 4° C with rabbit primary antibodies against Iba-1 (1:400 dilution, Wako Chemicals USA, Richmond, VA, USA). After washing with PBST, sections were incubated for 2 h at room temperature with biotinylated anti-rabbit secondary IgG (1:1000 dilution, Jackson ImmunoResearch, West Grove, PA, USA). Following PBS washes, sections were incubated with an avidin–biotin-peroxidase (ABC) complex (Vector Laboratories, Burlingame, CA, USA) for 45 min at room temperature. Staining was performed using 3,3′-diaminobenzidine (DAB) (Vector Laboratories, Burlingame, CA, USA). Thirteen images were captured at 40x magnification from three brain regions (cortex, hippocampus, and thalamus). Microglia were characterized as resting based on presence of ramification, with clearly observed processes and with relatively small cell bodies. Microglia were characterized as activated when cell bodies were larger with complex bushy processes or ameboid with round cell bodies without processes. [66, 67]. A shift to activated microglia state was observed with Ang II-induced hypertension. (Fig. S4)

**Figure S4. Microglial morphology shifts to an activated state in response to Ang II-induced hypertension.** Control mice exhibit a higher percentage of resting microglia compared to activated microglia, whereas Ang II-infused mice show the opposite trend. Data are presented as mean ± SEM, with n=3-5 per sex per group. ***p<0.001.
